# Does Insulin Like Growth Factor-1 (IGF-1) Deficiency Have a “Protective” Role in the Development of Diabetic Retinopathy in Thalassamia Major Patients?

**DOI:** 10.4084/MJHID.2015.038

**Published:** 2015-05-20

**Authors:** Vincenzo De Sanctis, Carlo Incorvaia, Ashraf T Soliman, Giancarlo Candini, Alessia Pepe, Christos Kattamis, Nada A. Soliman, Heba Elsedfy, Mohamed El Kholy

**Affiliations:** 1 Pediatric and Adolescent Outpatient Clinic, Quisisana Hospital, Ferrara, Italy; 2 Department of Ophthalmology, University of Ferrara, Ferrara, Italy.; 3 Department of Pediatrics, Division of Endocrinology, Alexandria University Children’s Hospital, Alexandria; 4 Medical Physicists, Honorary Member of Italian Association of Medical Physics ( AIFM ), Ferrara, Italy; 5 Cardiovascular MR Unit, Fondazione G. Monasterio CNR-Regione Toscana and Institute of Clinical Physiology, Pisa, Italy; 6 First Department of Paediatrics, University of Athens, Athens, Greece; 7 Ministry of Health, Alexandria, Egypt; 8 Department of Pediatrics, Ain Shams University, Cairo, Egypt

## Abstract

**Rationale:**

Both insulin and IGF-1 have been implicated in the control of retinal endothelial cell growth, neovascularization and diabetic retinopathy. Recent findings have established an essential role for IGF-1 in angiogenesis and demonstrated a new target for control of retinopathy that explains why diabetic retinopathy initially increases with the onset of insulin treatment

**Objective:**

This cross-sectional study was designed to give insights into relationship between Insulin-Growth-Factor 1 (IGF-1) levels and diabetic retinopathy (DR) in a sample of thalassemia major (TM) patients with insulin dependent diabetes mellitus (IDDM). This relation was not previously evaluated, despite the fact that both diseases co-exist in the same patient. The study also describes the clinical and biochemical profile of the associated complications in TM patients with and without IDDM.

**Design:**

A population-based cross-sectional study.

**Participants:**

The study includes 19 consecutive TM patients with IDDM and 31 age- and sex-matched TM patients without IDDM who visited our out-patient clinics for an endocrine assessment

**Methods:**

An extensive medical history, with data on associated complications and current medications, was obtained. Blood samples were drawn in the morning after an overnight fast to measure the serum concentrations of IGF-1, glucose, fructosamine, free thyroxine (FT4), thyrotropin (TSH) and biochemical analysis. Serologic screening assays for hepatitis C virus seropositivity (HCVab and HCV-RNA) were also evaluated; applying routine laboratory methods. Plasma total IGF-1 was measured by a chemiluminescent immunometric assay (CLIA) method. Ophthalmology evaluation was done by the same researcher using stereoscopic fundus biomicroscopy through dilated pupils. DR was graded using the scale developed by the Global Diabetic Retinopathy Group. Iron stores were assessed by direct and indirect methods.

**Results:**

Eighteen TM patients with IDDM (94.7 %) and ten non-diabetic patients (32.2 %) had IGF-1 levels below the 2.5^th^ percentile of the normal values for the Italian population. The mean serum IGF-1 concentrations were significantly lower in the diabetic versus the non-diabetic TM groups (p < 0.001). DR was present in 4 (21 %) of 19 TM patients with IDDM and was associated with the main classical risk factors, namely inefficient glycemic control and duration of the disease but not hypertension. Using the scale developed by the Global Diabetic Retinopathy Group, the DR in our patients was classified as non proliferative diabetic retinopathy (NPDR). Only a few numbers of microaneurysms [1–3] were detected. Our data also confirm the strong association of IDDM in TM patients with other endocrine and non-endocrine complications.

## Introduction

Insulin dependent diabetes (IDDM) and impaired glucose tolerance (IGT) are relatively common complications in thalassaemia major (TM) patients with iron overload and sub-optimal chelation therapy. The prevalence of IDDM and IGT in adolescents and young adults with TM mainly treated with desferrioxamine mesylate (DFO) varies in different studies from 0 to 21% and from 9.3 to 24.3 %, respectively. 1, 2

A substantial aim in diabetes care is the prevention, early detection and proper management of complications, including microvascular (diabetic retinopathy, nephropathy and neuropathy) and macrovascular complications (cardiovascular disease, cerebrovascular disease and peripheral vascular disease). Prevalence of diabetic retinopathy (DR) depends on various factors including age, sex (male), ethnicity, type of diabetes, pregnancy, hypertension, state of metabolic control and diabetes duration. 3,4

The prevalence of DR was examined in a population-based study in the Veneto region of North East Italy. Of 1321 diabetic patients selected, the prevalence of DR was 26.2% (24.4% background and 1.8% proliferative). The prevalence of DR was significantly related to the duration of diabetes (17.3% for less than five years; 60.8% for greater than 20 years). Proliferative retinopathy was much more prevalent after 20 years of diabetes. No significant differences were found in the prevalence of total or proliferative retinopathy between males and females.^4^

Numerous pathways have been implicated in the pathogenesis of DR. Hypoxia is one of the most important initiating factors. It is responsible for the activation of transcription factors such as hypoxia- inducible factor (HIF)-1 α and HIF-1 β; these factors, finally, bind to the hypoxia response elements of the vascular endothelial growth factor (VEGF) promoter. Another known modifier of VEGF expression is IGF-1.^5^

Studies on transgenic mouse models have shown the presence of retinal neovascularization associated with VEGF expression mediated by increased induction of IGF-1 in retinal glial cells.^6^

IGF-1 is known to trigger a critical cascade of molecular events that initiate retinal angiogenesis. Increased vitreous IGF-1 levels have been correlated with the severity of ischemia-associated diabetic retinal neovascularization.^7^–^11^ The action of IGF-1 may also depend on genetic factors and/or metabolic changes in the retinal epithelium affecting oxygenation, VEGF and P44/42 protein kinase activity. 7–11

Considering that IGF-1 administration to patients with diabetes improves diabetes control, by increasing insulin sensitivity and decreasing secondary GH resistance, Laron and Weinberger have speculated that IGF-1 has a ‘permissive’ mediating or even a ‘protective’ role in the development of diabetic retinopathy.^12^

This cross-sectional study was designed to give insights into the relationship between IGF-1 levels and DR in a sample of TM patients with IDDM. In none of the previous studies, this relation was evaluated, despite the fact that both diseases (DR and IDDM) coexist in the same patient. Furthermore, the study intends to describe the clinical and biochemical profile of the associated complications in TM patients with and without IDDM.

## Patients and Methods

### Setting and study design

The study was started at the beginning of 2009 by VDS, Coordinator of International Network of Clinicians for Endocrinopathies in Thalassemia and Adolescent Medicine (ICET-A)^13^ at the Thalassaemia Centre of Ferrara and was completed by the end of 2014 at the Quisisana Pediatric and Adolescent Outpatient Clinic of Ferrara.

The study included 19 consecutive TM patients with IDDM and 31 age- and sex-matched TM patients without evidence of IDDM, who visited our out-patient clinics for an endocrine assessment. The duration of diabetes was defined as the interval between diagnosis of IDDM and the time of enrollment in this study. Insulin usage was recorded for each diabetic patient.

### Exclusion criteria

Exclusion criteria were: 1) duration of diabetes less than 10 years; 2) previous or current treatment with drugs known to interfere with glucose or lipid metabolism or to influence blood pressure; 3) previous treatment with corticosteroids for longer than 2 weeks; 4) smoking of more than 15 cigarettes/day and alcohol abuse (more than three glasses of wine/day); 5) presence of factors that could interfere with fructosamine determination (such as low serum albumin concentration, dyslipidemia and hyperbilirubinemia).^14^

### Research design

An extensive medical history, including data on associated complications and current medications, was obtained and a physical examination including anthropometry (weight, height, BMI), vital signs (blood pressure, heart rate) and gonadal and menstrual status was performed. Body mass index (BMI) was calculated as the body weight divided by the height squared (Kg/m^2^). A subject was considered overweight when the BMI was between 25 and 29.9 and obese when the BMI was above 30.

The following clinical data were also recorded: age at first transfusion, duration, type and compliance of iron chelation therapy, compliance with treatment of diabetes, duration of diabetes and associated endocrine complications, as previously described.^15^ Subjects were considered to have a macrovascular disease if they had ever been diagnosed with a myocardial infarction or other vascular accident.

### Definitions

For the screening, diagnosis and treatment of growth disorders and endocrine complications we used the criteria as previously described. 15

### Blood sampling and methods

Blood samples were drawn in the morning after an overnight fast to measure the serum concentrations of IGF-1, glucose, fructosamine, free thyroxine (FT4), thyrotropin (TSH), urea, creatinine, electrolytes (including calcium and phosphate) and total proteins.

The last insulin injection on the day before blood sampling was administered at 22.00 h in IDDM subjects receiving intensive insulin therapy (four daily injections) and at 18.00 h in IDDM subjects receiving two daily injections of a mixture of short- and medium-acting insulins. TM patients were invited to abstain from ascorbic acid supplements for a minimum of 24 hours prior to sample collection.

In order to exclude severe liver injury and dysfunction, serum concentrations of alanine aminotransferase (ALT), gamma glutamyl transferase (γGT), alkaline phosphatase (ALP), total and direct bilirubin, albumin, prothrombin time (PT) and international normalization ratio (INR) were measured. Serologic screening assays for hepatitis C virus seropositivity (HCVab and HCV-RNA) were also obtained applying routine laboratory methods.

Plasma total IGF-1 was measured by a chemiluminescent immunometric assay (CLIA) method (Nichols Institute Diagnostics, San Juan, CA). The assay was performed after separation of IGF-1 from binding proteins by Liaison® autoanalyzer (DiaSorin SpA, Saluggia, Italy). The sensitivity of the test was six ng/ml, whereas the intra- and interassay coefficients of variation (CVs) of our in-house pooled serum control sample were 4.8% and 6.7 %, respectively.

The reported analytic sensitivity of this assay was from 6 to 25 ng/ml. Ranges of normal values set at the 2.5th–97.5th percentile in 547 non-hypopituitary, non-acromegalic healthy subjects of both sexes in Italy in three age ranges were: 95.6–366.7 ng/ml for ages 25 to 39 yrs, 60.8–297.7 ng/ml for 40 to 59 yrs and 34.5–219.8 ng/ml for subjects aged 60 and above.^16^ For the diagnosis of microalbuminuria, a first-morning urine sample was analyzed by immunoturbidimetry (MAU; normal range: 20–200 mg/l). 17

### Assessment of iron overload

Iron overload was assessed by direct and indirect methods. At the beginning of the study it was assessed by serum ferritin level and was arbitrarily categorized as mild (<1000ng/ml), moderate (1000–2000 ng/ml and severe (>2000ng/ml).^18^

In 10 TM patients with IDDM and 21 patients without IDMM, cardiac iron concentration (CIC) was available. Magnetic resonance imaging (MRI) was performed using a 1.5 T scanner (GE Signa/Excite HD, Milwaukee, WI, USA) within the Myocardial Iron Overload in Thalassemia (MIOT) network, where MRI scans are performed using homogeneous, standardized and validated procedures. 19 A conservative cut off value of T2^*^ > 20 ms was considered normal.^20^

The liver iron concentration (LIC) was assayed in 11 TM patients with IDDM and in 14 patients without IDDM by atomic absorption spectrophotometry and expressed as mg/g dry weight (dw) 21 or by MRI 22 or Superconducting Quantum Interference Device (SQUID) susceptometry. Liver T2^*^ values were converted into LIC values by using the calibration curve introduced by Wood et al.^23^ Based on data from the literature the normal LIC is considered between 0.4 and 2 mg/g of liver dry weight while iron overload is classified as mild: 2–7, moderate: 7–15 and severe > 15 mg Fe/gr dry wt.^23^

### Assessment of diabetic retinopathy (DR) and metabolic control

Nowadays, different standards for DR have been published, morphological features of lesions are commonly mentioned parameters for disease severity grading. 24–26 Ophthalmology evaluation was done by the same researcher (CI) using stereoscopic fundus biomicroscopy through dilated pupils. DR was graded using the scale developed by the Global Diabetic Retinopathy Group (Table 1).^25^ When the diabetic retinopathy was asymmetric, the subject was assigned to the group corresponding to the eye with the worse retinopathy findings.

In each case, metabolic glucose control was assessed by the concentration of fasting blood glucose, the serum concentration of fructosamine and the results of glycosuria and ketonuria monitoring at home. Poor control was arbitrarily defined as the presence of a fasting blood glucose concentration >11.1 mmol/l and/or a serum concentration of fructosamine > 350 μmol/l.

### Ethical aspects

The study protocol was conducted in accordance with the ethical guidelines of the 1996 Declaration of Helsinki. 27 Informed consent was obtained from all patients.

### Statistical Analysis

Characteristics of the studied patients are reported as total number and mean ± standard deviation (SD). Statistical significance of the differences between variables was assessed using the unpaired two-tailed Student’s t test. Fisher’s Exact test was used to calculate the probability value for the relationship between two dichotomous variables. A p value < 0.05 was considered as significant. A software program used for the statistical analysis was developed by Dr. Candini (Department of Medical Physics, St. Anna Hospital, Ferrara, Italy) and validated according to Alder and Roesser.^28^

## Results

### Patients’ characteristics

All patients were on regular transfusions (mean haemoglobin level 11.5 g/dl) and iron chelation therapy with deferoxamine (43 patients: 30–45 mg/kg body weight, 4–6 days a week by slow subcutaneous infusion by pump, starting in 1977–1978), or oral deferiprone (26 patients: 75 mg/kg body weight daily), or deferiprone plus deferoxamine (12 patients; 75 mg/kg body weight daily and 40 mg/kg body weight, 3 days a week, by slow subcutaneous pump infusion) or oral deferasirox (6 patients: 20–30 mg/kg body weight daily). Chelation therapy has changed over time. Treatment with intramuscular deferoxamine has been available for most patients since 1969. Regular subcutaneous infusion of deferoxamine was started in 1978. Since 1995 and 2007 the new oral chelating agents, deferiprone or deferasirox, have been given to some patients who were unable or unwilling to receive deferoxamine. Deferiprone plus deferoxamine was given to few selected patients with severe iron overload.

The baseline clinical characteristics of 19 IDDM subjects with TM (7 women and 12 men) are shown in Table 2. All TM patients with IDDM were on an insulin dose schedule of 2–4 times daily, using combinations of short-acting and intermediate-acting insulin. Age, gender distribution and BMI did not differ between diabetic and non-diabetic patients. One diabetic patient was overweight, and one was obese. All patients had normal systolic and diastolic blood pressures.

A significant percentage of TM patients with IDDM had other endocrinopathies (Table 1). Hypogonadotropic hypogonadism was present in 89.5% of diabetic versus 54.8% of non diabetic patients (p <0.05). All but five were on hormone replacement therapy with sex steroids. Hypothyroidism and hypoparathyroidism were significantly more prevalent in diabetic versus non-diabetic patients, but statistically different only for hypothyroidism (p <0.05). All hypothyroid and hypoparathyroid TM patients were taking thyroxine or calcitriol respectively. No adrenal insufficiency was documented. Growth hormone status was not assessed.

An abnormal ALT value (> 80 U/L) was observed in 4 female TM patients with IDDM (21 %) and in 2 (1male and 1 female) TM patients without IDDM (6.4 %). A statistical difference was observed for serum γGT in the two groups (p< 0.05). Hepatitis C antibodies were present in 100 % in both groups (Table 1). The percentage of HCV-RNA seropositivity did not differ in diabetic versus non-diabetic patients (57.9% versus 51.6%).

Serum lipids were not significantly altered in the two groups of patients compared to the normal ranges of our central laboratory service (< 180 mg/dL for total cholesterol; < 159 mg/dL for LDL-C and 150 mg/dL for triglycerides). Only one male TM patient with DR had a borderline total cholesterol level (179 mg/dL). Two patients had triglyceride levels higher than 130 mg/dL. Ten of our patients with DR had low HDL-C levels (<40 mg/dL for men and <50 mg/dL for women) and only one patient had LDL-C level higher than 159 mg/dL (168 mg/dL).

### Assessment of iron overload

Mean serum ferritin level was significantly higher in diabetic versus non-diabetic TM subjects (p <0.05). Serum ferritin level >2000 ng/ml (severe iron overload) was present in 15.8% of diabetic versus 3.2% of non-diabetic TM patients.

A liver iron concentration > 7 mg/g dry weight was present in 15.8 % of diabetic TM patients and none of the non-diabetic TM patients. A 36 year old female patient with IDDM had a LIC concentration of 15.9 mg/g/dw (a concentration associated with a high risk for cardiac disease). Although the percentage of TM patients with T2^*^ < 20 msec was higher in IDDM compared to those without diabetes, the difference was not statistically significant (Table 2).

### Insulin-like growth factor 1 (IGF-1) analysis

Eighteen TM patients with IDDM (94.7 %) and 10 non-diabetic patients (32.2 %) had IGF-1 levels below the 2.5^th^ percentile of the normal values for the Italian population. 16 The mean serum IGF-1 concentrations were significantly lower in the diabetic versus the non-diabetic TM groups (p < 0.001; Table 2).

**Figure 1:**
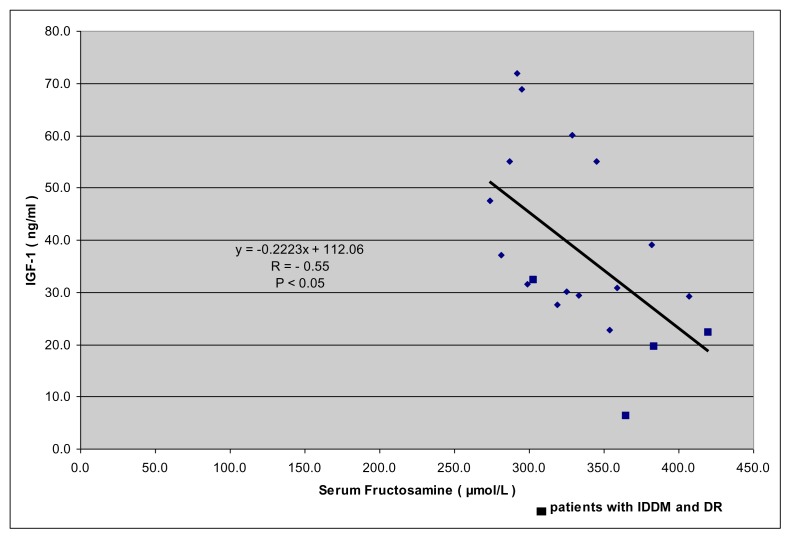
Correlation between fructosamine (μmol/l) and IGF-1 (ng/ml). *Normal IGF-1 values: 95.6–366.7 ng/ml for ages 25 to 39 yrs, 60.8–297.7 ng/ml for 40 to 59 yrs and 34.5–219.8 ng/ml. 16* Assessment of diabetic retinopathy (DR) and metabolic control.

A significant negative correlation was observed between IGF-1 and fructosamine levels in diabetic and non diabetic TM patients (Figure 1; r: − 0.5516.; p: < 0.05).

In our study, DR was present in a low, percentage in 4 (21 %) of 19 TM patients with IDDM and was associated with the main classical risk factors, of inefficient glycemic control and duration of disease but not of hypertension (Table 3). Using the scale developed by the Global Diabetic Retinopathy Group^25^, the DR in our patients was classified as non proliferative diabetic retinopathy (NPDR). Only a few numbers of microaneurysms (1–3) were detected. Certain recognized ocular and systemic factors which are known to protect a diabetic patient against the development of DR, such as high myopia, raised intraocular pressure and moderate carotid stenosis were not present or reported in our TM patients.

In eight diabetic patients inefficient metabolic glucose control, arbitrarily defined as the presence of a fasting blood glucose concentration > 11.1 mmol/l and/or a serum concentration of fructosamine > 350 μmol/l, was found (Fig 1). Microalbuminuria was present in five patients with IDDM (26.3%).

In two patients with fair (no.1) and good (no.3) compliance to combined iron chelation therapy, the LIC (mg Fe/gr dry wt) was normal (Table 3).

## Discussion

IDDM is a complex disease with many end organ complications. However, good control of the disease can prevent the onset or delay the progression of the various complications, including diabetic retinopathy (DR). The prevalence of DR worldwide ranges from 6.8 to 44.4% in patients with diabetes mellitus.^29^–^31^

A systematic literature review was conducted by Yau et al. to identify all population-based studies in general populations or individuals with diabetes. A total of 35 studies (1980–2008) provided data from 22,896 individuals with diabetes. The overall prevalence was 34.6% for any DR, 6.96% for proliferative DR, 6.81% for diabetic macular edema, and 10.2% for vision-threatening diabetic retinopathy (VTDR). All DR prevalence end points increased with diabetes duration, hemoglobin A_1c_, and blood pressure levels were higher in people with type 1 compared with type 2 diabetes. 29 Similar results were also reported by other Researchers.^30^–^32^

Our screening modality of DR was in accordance with the UK National Institute for Clinical Excellence (NICE) recommendations regarding the sensitivity, specificity and technical failure rate. 33 In our study, DR was present in a relatively small percentage (21 %) of diabetic TM patients with duration of diabetes above ten years. IDDM was associated with the main classical risk factors of inefficient glycemic control and duration of the disease but not of hypertension (Table 3). Using the scale developed by the Global Diabetic Retinopathy Group 25, DR in our patients was classified as non proliferative diabetic retinopathy (NPDR). A small number of microaneurysms (from 1 to 3) were detected by an expert ophthalmologist using a stereoscopic fundus biomicroscopy through dilated pupils.

The major risk factors for DR are hyperglycemia, hypertension, duration of diabetes and dyslipidemia. Genetic and growth factors have also been implicated in the development of DR. On the other hand, there are certain recognized systemic and ocular factors which are known to protect a diabetic patient against the development of DR, e.g. low lipid levels, hypogonadism, growth hormone deficiency, reduced IGF-1 levels, high myopia, raised intraocular pressure and moderate carotid stenosis.^34^–^41^

In our TM patients serum lipids were within the defined normal range (< 180 mg/dL for total cholesterol; < 159 mg/dL for LDL-C and 150 mg/dL for triglyceride).

Several mechanisms may play a role in determining low serum cholesterol and triglyceride levels in TM patients including plasma dilution resulting from anemia, increased cholesterol requirement associated with erythroid hyperplasia, macrophage system activation with cytokine release, increased cholesterol uptake by the reticuloendothelial system, iron overload and oxidative stress. In the light of these data, it would be speculated that the low prevalence of DR in TM patients compared to patients with diabetes mellitus might be a consequence of a better lipid profile.

All male TM patients but one with hypogonadism were on hormone replacement therapy with long acting sex steroids. Their serum free testosterone levels, however, were in the subnormal or below the adult normal range because of the presence of a high serum SHBG level secondary to chronic liver disease and iron overload. Therefore, a mild degree of androgen insufficiency could be an additional factor in reducing the risk of DR in TM patients.

Unfortunately, growth hormone was not assessed routinely in our patients because the majority of them were followed in an outpatient endocrine clinic. Nevertheless, data from the literature report a prevalence of GHD and/or IGF-I deficiency in TM patients from 8% to 44 % in different centers.^15^–^18^ It has been reported that IGF-1 is a potent stimulator of retinal endothelial cell growth and play a major role in the development of diabetic retinopathy.

A significant number of our TM patients with and without IDDM had an IGF-1 deficiency (< 2.5^th^ percentile of age- and sex- matched Italian population). However, the mean IGF-1 value was particularly lower in diabetic versus non-diabetic patients (p < 0.001). Decreased IGF-1 secretion occurs in the majority of TM patients especially those with growth and pubertal delay. Many factors contribute to this decreased synthesis of IGF-1 including under-nutrition, insufficient blood transfusion with significant periods of anemia, inadequate iron chelation with iron overload in the pituitary gland (GH, LH, FSH, TSH deficiencies), liver (systemic IGF-1 deficiency) and the co-occurrence of other endocrine disorders, such as hypothyroidism and diabetes mellitus. 42–44 IDDM group was significantly more iron overloaded than nondiabetic group as indicated by the higher serum ferritin and LIC values.

Our results call into question whether very low serum IGF-1 contributes to the pathogenesis of DR, inhibiting the molecular events that initiate retinal angiogenesis. The pathogenesis of diabetic retinopathy is complex. The major causative factor for the development of diabetic retinopathy is hyperglycemia, which leads to increased vasopermeability, endothelial cell proliferation, and neovascularisation. While hyperglycemia is a major factor, diabetes is associated with changes in insulin, IGF-1 and many other hormones and metabolites, including free fatty acid, amino acids, advanced glycation end products, and components of the oxidative stress pathway. IGF-1 is known to trigger a critical cascade of molecular events that initiate retinal angiogenesis. 45

The proliferative effect of hyperglycemia on vascular endothelial cells is thought to be mediated by vascular endothelial growth factor (VEGF). 46 IGF-1 receptor regulation of VEGF action is mediated at least in part through control of VEGF activation of p44/42 mitogen-activated protein kinase, establishing a hierarchical relationship between IGF-1 and VEGF receptors. Therefore, these findings establish an essential role for IGF-1 in angiogenesis and demonstrate a new target for control of retinopathy and also explain why diabetic retinopathy initially increases with the onset of insulin treatment. 47–49

Nevertheless, while experimental and clinical evidence suggests that serum IGF-1 concentrations may be involved in the development of diabetic retinopathy the relationship is still controversial.

Several studies have reported that higher serum IGF-1 levels may be a risk factor for the development of severe diabetic retinopathy; on the other hand few studies have shown no association between serum IGF-1 levels and the development or progression of diabetic retinopathy.

In our patients, a significant correlation was observed between fructosamine levels and IGF-1 values. It has been reported by Chantelau that the reduction of hyperglycaemia from >16 mmol/l (equivalent to HbA_1c_ >11%) to <10 mmol/l (HbA_1c_ <8%) increase the serum IGF-1 levels by 70–220%, within 5 months.^45^ While proteinuria and symptomatic neuropathy regressed in his patients, retinopathy progressed from the mild to the severe non-proliferative stage with maculopathy (n=4), and to the proliferative stage (n=1). The biological mechanisms underlying this phenomenon remain unknown. Insulin signalling in endothelial cells has been shown to regulate the expression of some potential mediators of neovascularization, including VEGF, eNOS and endothelin-1.^35^–^41^

These findings are probably less relevant in TM patients because the IGF-1 levels are < −2SDs in 50% of patients compared to healthy individuals 44 and the prevalence of DR remains low for a long duration of diabetes, however, we cannot ignore this point because it suggests that another particular risk factor for the progression of DR is represented by the upregulation of serum IGF-1. 46–47

We used fructosamine as an index of metabolic control because a number of clinically significant haemoglobin disorders alter haemoglobin, structurally or chemically, thereby affecting the reliability of the A1c test. 50 As the mean half-life of plasma proteins is approximately 2–3 weeks, fructosamine provides a shorter term representation of glycaemic control than HbA1c. However, it should be noted that a recent clinical trial in patients with TM has shown a direct correlation between fructosamine and fasting blood glucose values.^51^

Our data confirm the strong association of IDDM in TM patients with other endocrine and non-endocrine complications related to severe iron overload. Therefore, early recognition of these complications, establishment of appropriate chelation therapy and adequate treatment of each complication are the main keys to successful management of these patients

## Conclusions

This study although not fully representative of the general population suggests a possible ‘protective’ role of low IGF-1 in the development of diabetic retinopathy. Therefore, it could be interesting to study further possible systemic, local and genetic factors in relation to the development and degree of DR in a larger cohort of patients with diabetes versus diabetic TM patients. Although specific guidelines have been prepared and published by the ICET-A Network for the treatment of endocrine complications in thalassemic patients 52,53, there is still an urgent need to consider specific worldwide treatment protocols for managing patients with multiple/complex complications.

## Figures and Tables

**Table 1:** Diabetic Retinopathy Disease Severity Scale. Global Diabetic Retinopathy Group^25^

Proposed Disease Severity Level	Findings Observable on Dilated Ophthalmoscopy
No apparent retinopathy	No abnormalities
Mild nonproliferative diabetic retinopathy	Microaneurysms only
Moderate nonproliferative diabetic retinopathy	More than just microaneurysms but less than severe nonproliferative diabetic retinopathy
Severe nonproliferative diabetic retinopathy	Any of the following: more than 20 intraretinal hemorrhages in each of 4 quadrants; definite venous beading in 2 quadrants; Prominent intraretinal microvascular abnormalities in 1 quadrant and no signs of proliferative retinopathy
Proliferative diabetic retinopathy	One or more of the following: neovascularization, vitreous/preretinal hemorrhage

**Table 2:** Demographic, clinical and laboratory features of a study population of 50 adults with thalassemia major, 19 patients with insulin dependent diabetes mellitus (IDDM) and 31 without IDDM

Variables	DM	No-DM	p-value
	(N= 19)	(N= 31)	
Sex (M/F)	12/7	10/21	NS
Age (yrs)	42.1 ± 4.9	40.4 ± 3.5	NS
BMI, kg/m^2^	22.9 ± 4.7	22.2 ± 2.9	NS
Diabetes duration (years)	20.7 ± 3.2	NA	NA
HCV Ab (%)	100	100	NS
HCV-RNA (%)	57.9	51.6	NS
ALT (U/L)°	52.1 ± 48.6	36.3 ± 32.4	NS
γ GT (U/L)°°	53.8 ± 64.9	21.1 ± 10.6	< 0.05
Serum ferritin (ng/ml)	1169.4 ± 943.5	744.1 ± 548.6	< 0.05
IGF-1 (ng/ml)	37.7 ± 17.5	65.4 ± 4.5	< 0.001
Hypogonadism (%)	89.5	54.8	< 0.05
Hypothyroidism (%)	47.4	16.1	< 0.05
Hypoparathyroidism (%)	21.1	9.7	NS
Global Heart T2* < 20 msec (%)	40*	9.5**	NS
LIC > 7 mg/g dry weight (%)	15.8+	0	NA

Legend:

° Alanine aminotransferase;

°° gamma glutamyl transferase;

* 4/10 TM patients;

** 2/21 TM patients;

+ 11/19 TM patients;

NA: not applicable.

**Table 3:** Demographic, clinical and laboratory features of thalassemia major with IDDM and DR.

Patient (No.)	Age (yrs)	BMI (kg/m^2^)	Diabetes duration (years)	IGF-1 (ng/ml)	FRT (μmol/L)	ALT (U/L)	γ-GT (U/L)	Serum ferritin (ng/ml)	LIC mg/g dw	Global Heart T2* msec	LEF (%)
1	48.1	18.4	29	6.2*	365	65	68	1230	0.9	55	70
2	35.5	23.4	15	19.5	384 plus Mab	87	33	2210		7	68
3	44.9	24.9	25	22.3	420	23	34	657	0.7	39	66
4	36.4	23.0	20	32.3	303 plus Mab	104	147	3665	15.8	13	69

Legend: BMI: body mass index; FRT: fructosamine; Mab: microalbuminuria; ALT: alanine aminotransferase; γ-GT: gamma glutamyl transferase; LIC: liver iron concentration; LEF: left ventricular ejection fraction assessed by echography;

* TM patient with severe chronic liver disease
